# Invasive Non-typhoidal *Salmonella* Infections in Asia: Clinical Observations, Disease Outcome and Dominant Serovars from an Infectious Disease Hospital in Vietnam

**DOI:** 10.1371/journal.pntd.0004857

**Published:** 2016-08-11

**Authors:** Nguyen Phu Huong Lan, Tu Le Thi Phuong, Hien Nguyen Huu, Le Thuy, Alison E. Mather, Se Eun Park, Florian Marks, Guy E. Thwaites, Nguyen Van Vinh Chau, Corinne N. Thompson, Stephen Baker

**Affiliations:** 1 The Hospital for Tropical Diseases, Ho Chi Minh City, Vietnam; 2 The Hospital for Tropical Diseases, Wellcome Trust Major Overseas Programme, Oxford University Clinical Research Unit, Ho Chi Minh City, Vietnam; 3 Centre for Tropical Medicine, Nuffield Department of Clinical Medicine, Oxford University, Oxford, United Kingdom; 4 Hawaii Center for AIDS, University of Hawaii at Manoa, Honolulu, Hawaii, United States of America; 5 Department of Veterinary Medicine, the University of Cambridge, Cambridge, United Kingdom; 6 International Vaccine Institute, Seoul, Republic of Korea; 7 The London School of Hygiene and Tropical Medicine, London, United Kingdom; University of Tennessee, UNITED STATES

## Abstract

Invasive non-typhoidal *Salmonella* (iNTS) infections are now a well-described cause of morbidity and mortality in children and HIV-infected adults in sub-Saharan Africa. In contrast, the epidemiology and clinical manifestations of iNTS disease in Asia are not well documented. We retrospectively identified >100 cases of iNTS infections in an infectious disease hospital in Southern Vietnam between 2008 and 2013. Clinical records were accessed to evaluate demographic and clinical factors associated with iNTS infection and to identify risk factors associated with death. Multi-locus sequence typing and antimicrobial susceptibility testing was performed on all organisms. Of 102 iNTS patients, 71% were HIV-infected, >90% were adults, 71% were male and 33% reported intravenous drug use. Twenty-six/92 (28%) patients with a known outcome died; HIV infection was significantly associated with death (*p* = 0.039). *S*. Enteritidis (Sequence Types (ST)11) (48%, 43/89) and *S*. Typhimurium (ST19, 34 and 1544) (26%, 23/89) were the most commonly identified serovars; *S*. Typhimurium was significantly more common in HIV-infected individuals (*p* = 0.003). Isolates from HIV-infected patients were more likely to exhibit reduced susceptibility against trimethoprim-sulfamethoxazole than HIV-negative patients (*p* = 0.037). We conclude that iNTS disease is a severe infection in Vietnam with a high mortality rate. As in sub-Saharan Africa, HIV infection was a risk factor for death, with the majority of the burden in this population found in HIV-infected adult men.

## Author Summary

Invasive non-typhoidal *Salmonella* (iNTS) infections occur when *Salmonella* bacteria, which normally cause diarrhea, enter the bloodstream and spread through the body. Invasive NTS infections have become a common cause of infection and death in children with malaria and adults with HIV in sub-Saharan Africa. However, it is unknown whether iNTS is as common or as severe outside sub-Saharan Africa. We evaluated over 100 iNTS cases from an infectious disease hospital in southern Vietnam admitted between 2008–2013. We used hospital records to determine the clinical features of iNTS disease and to identify risk factors associated with death and performed typing of the isolated organisms. The majority of patients were HIV positive (72/102, 71%), >90% of patients were adults, 71% were male and 33% reported intravenous drug use. The mortality rate of iNTS patients was 28% (26/92), and HIV infection was a significant risk factor for fatal outcome (*p* = 0.039). The serovars most commonly identified were *S*. Enteritidis and *S*. Typhimurium; *S*. Typhimurium was found more frequently in HIV-positive individuals (*p* = 0.003). We report that iNTS disease is a severe infection in Vietnam with a high mortality rate. Similar to sub-Saharan Africa, HIV infection was a strong risk factor for death.

## Introduction

Infections with organisms belonging the bacterial genus *Salmonella* are associated with a range of disease syndromes in humans. A small subset of the >2,500 described serovars of that belong to *Salmonella* subspecies I are capable of causing typhoidal illness, these include *Salmonella enterica* serovar Typhi (*S*. Typhi) and the various *S*. Paratyphi pathovars [1]. However, the vast majority of the *Salmonella* subspecies I serovars are not commonly associated with systemic disease in humans and are referred to as non-typhoidal *Salmonella* (NTS). NTS organisms include *S*. Typhimurium, *S*. Dublin and *S*. Enteritidis, which are characterized by their wide host range and their ability to induce a self-limiting diarrhea [2]. However, in addition to the common diarrheal clinical syndrome induced by NTS organisms in humans, invasive (bloodstream) NTS (iNTS) disease can also occur in specific populations [3,4]. iNTS disease, which is most commonly caused by the *Salmonella* serovars Typhimurium and Enteritidis [5,6], is associated with an aggressive systemic infection that loosely resembles typhoid fever [2,4,7]. In sub-Saharan Africa, the disease has a high mortality rate (20–25%) and infection is most common in children with malaria, malnourished children and HIV-infected adults [3]. There are an approximately 1.9 million cases of iNTS disease in sub-Saharan Africa annually, with an overall estimated incidence rate of 227 per 100,000 population [8], and 175–388 and 2,000–7,500 per 100,000 population in children 3–5 years of age and HIV-infected individuals, respectively [3,9–14].

NTS are a common cause of diarrhea in Asia, and we have previously shown that NTS are responsible for approximately 4% of pediatric hospitalized diarrhea in Ho Chi Minh City (HCMC), Vietnam [15]. In a retrospective study of blood cultures conducted between 1994 and 2008 at the Hospital for Tropical Diseases (HTD) in HCMC we observed that *S*. Typhi was the predominant cause of culture positive bacteremia (66%) until 2002 [16]. After this period we detected a significant annual decline in the isolation rate of *S*. Typhi and a concurrent increase in organisms associated with the HIV epidemic, including NTS. The isolation rate of NTS increased from 1% (n = 47) of total bacteremia cases between 1994–2001 to 4% (n = 146) of cases from 2002–2008. Whilst the increase in burden was modest in comparison to sub-Saharan Africa these data support a longitudinal shift in the etiology of bloodstream infections in southern Vietnam.

There is a paucity of data regarding iNTS infections from Asia, with limited reports from Taiwan [17], India [18], Thailand [19,20] and our aforementioned study in Vietnam [16]. It is apparent that the burden of iNTS in sub-Saharan Africa is not mirrored in Asia. However, iNTS disease is present in Asia but there are no or few data regarding clinical symptoms, disease outcome, patient demographics or the infecting serovars. By accessing available clinical data and bacterial isolates we sought to retrospectively investigate the clinical and microbiological manifestations of iNTS in a major infectious disease hospital in southern Vietnam.

## Materials and Methods

### Ethics statement

Ethical approval for this study was provided by the institutional review board (IRB) of the HTD. This study was performed retrospectively with routine anonymous laboratory and clinical data; individual patient identifying were not accessed and informed consent was not required.

### Study setting and population

HTD is a 550-bed hospital that serves as a main primary and secondary facility for the surrounding local population in HCMC and a tertiary referral center for infectious diseases for the southern provinces of Vietnam. Nearly 70% of HTD admissions live in HCMC, with the remainder residing in the surrounding provinces. Neonates, patients without infectious diseases, including those with surgical requirements, tuberculosis, cancer, primary hematological disorders or immunosuppression (other than HIV) are referred to other hospitals within HCMC. HIV-infected children are often referred to local pediatric hospitals.

The study population consisted of all individuals from which an NTS organism was isolated alone or in combination with an additional pathogen in blood culture from January 2008 through June 2013. This source data was collected from routine microbiology laboratory logbooks in which data from positive and negative blood culture are recorded. Patients with multiple positive blood cultures for the same NTS serogroup and antimicrobial susceptibility profile were considered to be a single case.

### Data collection and definition of disease outcome

A patient record form was used to collect clinical and laboratory data from the hospital chart for every patient. Clinical data recorded on admission included sex, HIV status (HIV diagnosed according to the World Health Organization (WHO) guidelines [21]), axillary temperature, presence of co-infection and hospital outcome. Outcome was classified based on clinician notes as follows: (1) recovery or improvement, (2) worsening status on discharge (often deteriorating patients taken from hospital by their relatives to die at home—a common custom in Vietnam), (3) death or (4) transfer to a different hospital (patient’s condition was unchanged but transferred to other hospital for specific treatment or surgery intervention, or patient left against medical advice). Outcome 2 and outcome 3 were considered to be fatal. Laboratory data was comprised of standard hematology and biochemical testing from hospital records on the day of admission.

### Statistical analysis of clinical and laboratory data

Clinical and laboratory data were compared between groups using Fisher's exact or Kruskal-Wallis tests for categorical and continuous data, respectively. We performed univariable and multivariable logistic regression to evaluate covariates that were independently associated with fatal outcome. Covariates selected for multivariable analysis *a-priori* included age, sex and immunosuppression (HIV status, hepatitis), in addition to other fixed demographic or clinical covariates that were significantly associated (*p*<0.05) with outcome from the univariate analysis. All statistical analyses were performed using Stata version 11 (StataCorp, College Station, TX, USA).

### Microbiological procedures

For blood culturing, 5–10 mL of venous blood from adults and 2–5 mL of venous blood from infants and children was inoculated into BACTEC plus aerobic bottles (Becton Dickinson). Inoculated BACTEC bottles were incubated at 37°C in a BACTEC 9050 automated analyzer for up to five days and sub-cultured when the machine indicated a positive signal. Organisms were identified by standard methods including API20E identification kits (Bio-Mérieux, Craponne, France). Specific grouping antisera were used to identify the serogroup of the isolated *Salmonella* on original culture. Vi antisera (along with 0:9) was used to identify *S*. Typhi; these were excluded from all analyses. All NTS isolated from blood cultures were stored in Brain Heart Infusion (BHI) glycerol at -70°C. For the purposes of this study all NTS isolated were recovered on MacConkey agar and subjected to re-identification and antimicrobial susceptibility testing. Re-identification of *Salmonella* serogroups was performed using specific grouping antisera as before. Antimicrobial susceptibility testing was performed on Muller-Hinton agar against ampicillin, amoxicillin/clavulanate, azithromycin, ceftazidime, ceftriaxone, chloramphenicol, ciprofloxacin, gentamicin, nalidixic acid, ofloxacin and trimethoprim-sulfamethoxazole, using the disk diffusion method as recommended by CLSI guidelines [22]. Antimicrobial disks were purchased from Oxoid (Thermo Fisher Scientific, UK) and susceptibility was determined using the Clinical Laboratory Standard Institute (CLSI) guidelines [22].

To further characterize the iNTS isolates, all organisms were genotyped (and molecular serotyped) using multi-locus sequence typing (MLST) following previously described methods [23,24]. Briefly, a set of seven housekeeping genes (*aroC*, *dnaN*, *hemD*, *hisD*, *purE*, *sucA* and *thrA*–primer sequences accessed at http://mlst.warwick.ac.uk/mlst/dbs/Senterica) were PCR amplified using template DNA extracted from each isolate after boiling bacterial colonies in PBS. PCR amplicons were cleaned using Agentcourt Ampure XP (Beckman Coulter) and were sequenced in both directions using BigDye Terminator v3 (Applied Biosystems, USA) followed by capillary sequencing on a 3130XL Genetic Analyzer (Applied Biosystems, USA). All sequences were manually trimmed to align to a reference sequence and were submitted to the previously mentioned MLST database for allelic profile and molecular serotyping (i.e. inferring serovar from MLST profile). A minimum spanning tree was created using the allelic profiles (variation in number of alleles between isolates of the seven housekeeping genes) using Bionumerics software (Applied Mathematics).

## Results

### The demographic and laboratory features of invasive non-typhoidal *Salmonella* infections

Between January 2008 and June 2013 there were 142 culture confirmed bloodstream infections caused by an NTS bacterium in this single centre. Hospital records were obtainable for 102/142 (72%) iNTS cases. The median patient age was 33 years (IQR: 28 to 41 years) (Table 1). Eight of the 102 (8%) iNTS cases were children (<16 years) of which five (5% of total) were infants (<12 months). The majority of patients (61/102; 60%) were from HCMC, with the remainder residing in the surrounding provinces. The median duration of illness (including fever and other symptoms) prior to hospital admission was 13 days (IQR 1–60 days). Patients were more commonly male (72/102; 71%) and three quarters (71/94; 76%) of adults (>16 years) reported that they were unemployed upon admission. A third (31/102; 33%) of cases reported a history of intravenous drug use, which was more common in men (26/65, 40%) than women (5/30, 17%) (*p* = 0.019, Fisher’s exact test).

**Table 1:** The clinical characteristics of invasive non-typhoidal *Salmonella* disease stratified by HIV infection status and outcome.

Characteristic	Total	HIV infection status		Fatal ^a^	
		Infected	Uninfected	Yes	No
	n = 102	n = 72	n = 30	n = 26	n = 66
Male sex	72 (70.6)	54 (75.0)	18 (60.0)	18 (69.2)	44 (66.7)
Age	33 (28–41)	31.5 (28–37)	44 (25–69)	33.5 (29–37)	33 (27–44)
Reported IDU	31/95 (32.6)	31/65 (47.7)	0 (0)	12/25 (48)	15/60 (25)
**Immunosuppression**					
HIV infection	72 (70.6)	72 (100)	0 (0)	24 (92.3)	39 (59.1)
On ART (HIV infected)	16/72 (22.2)	16/72 (22.2)	0/20 (0)	8/24 (33.3)	7/39 (17.9)
Hepatitis	27 (27.0)	20/70 (28.6)	7 (23.3)	8/25 (32.0)	17 (25.8)
**Symptoms and signs on admission**					
Abdominal pain	23 (22.5)	14 (19.4)	9 (30.0)	5 (19.2)	17 (25.8)
Ascites	8 (7.8)	8 (11.1)	0 (0)	2 (7.7)	4 (6.1)
Cough	44 (43.1)	35 (48.6)	9 (30.0)	14 (53.8)	26 (39.4)
Diarrhea	42 (41.2)	28 (38.9)	14 (46.7)	11 (42.3)	26 (39.4)
Fever	76/97 (78.4)	49/69 (71)	27/28 (96.4)	15/24 (62.5)	54/63 (85.7)
Hepatomegaly	25 (24.5)	21 (29.2)	4 (13.3)	4 (15.4)	18 (27.3)
Oral candidiasis	36 (35.3)	35 (48.6)	1 (3.3)	11 (42.3)	21 (31.8)
Pallor	56/101 (55.4)	38/71 (53.5)	18 (60.0)	15 (57.7)	35 (53.0)
Pneumonia	72 (70.6)	56 (77.8)	16 (53.3)	19 (73.1)	45 (68.2)
Septic Shock	6 (5.9)	4 (5.6)	2 (6.7)	4 (15.4)	2 (3.0)
Splenomegaly	16 (15.7)	15 (20.8)	1 (3.3)	3 (11.5)	10 (15.2)
Tachypnea	29 (28.4)	19 (26.4)	10 (33.3)	13 (50.0)	15 (22.7)
Throat lesions	45 (44.1)	40 (55.6)	5 (16.7)	13 (50.0)	26 (39.4)
**Coinfections**	16 (15.7)	16 (22.2)	2 (6.7)	6 (23.1)	10 (15.2)
*T*. *marnefeii*	9 (8.8)	9 (12.5)	0 (0)	2 (7.7)	7 (10.6)
Other ^b^	7 (6.9)	7 (9.7)	0 (0)	4 (15.4)	3 (4.5)
**Serovar**					
*S*. Enteritidis	43/89 (48.3)	30/63 (47.6)	13/26 (50.0)	12/22 (54.5)	26/57 (45.6)
*S*. Typhimurium	23/89 (25.8)	22/63 (34.9)	1/26 (3.8)	7/22 (31.8)	11/57 (19.3)
*S*. Choleraesuis	11/89 (12.4)	9/63 (14.3)	2/26 (7.7)	1/22 (4.5)	10/57 (17.5)
Other	12/89 (13.5)	2/63 (3.2)	10/26 (38.5)	2/22 (9.1)	10/55 (18.2)

^a)^ 10 patients missing outcome status;

^b)^ Includes *Cryptococcus neoformans*, *Streptococcus pneumoniae* and *Escherichia coli* isolates cultured from either blood or cerebrospinal fluid

HIV testing was performed for all patients diagnosed with iNTS infections; CD4 counts were not routinely measured. Seventy-two (71%) of the iNTS cases were HIV-infected: 71 adults (76% of 94 adults) and one infant (13% of all 8 children). Only 16/72 (22%) of the adult HIV-infected iNTS patients were on active antiretroviral therapy (ART) prior to this episode of bacteremia, and 6/72 (8%) of the HIV-infected iNTS patients were taking trimethoprim-sulfamethoxazole for *Pneumocystis jiroveci* pneumonia prophylaxis on admission. A history of long-term steroid use was reported in 4/30 (13%) of the iNTS cases testing negative for HIV infection.

Table 1 describes the clinical characteristics of the patients. The most common clinical features were fever (76/97, 78%) (≥38.0°C) and pallor (56/101, 56%). Almost half of the cases (45/102) had oralpharyngeal lesions, including ulcers and candidiasis; these symptoms were chiefly restricted to the HIV-infected group. Notably, gastrointestinal symptoms such as diarrhea (42/102, 41%) and abdominal pain (23/102, 23%), which are synonymous with the archetypal, non-invasive manifestation of NTS infection, were uncommon. However, comorbidities such as hepatitis (induced by hepatitis B, C or alcohol abuse) and pneumonia (caused by PCP or *Mycobacterium tuberculosis*) were recorded in 27% (27/102) and 71% (72/102) of patients, respectively. Furthermore, 16/102 (16%) patients had an additional pathogen identified in their bloodstream (BS) or cerebrospinal fluid (CSF): 9 *Talaromyces marnefeii* (BS), 4 *Cryptococcus neoformans* (CSF), 2 *Escherichia coli* (BS) and 1 *Streptococcus pneumoniae* (BS). None of these additional BS or CSF infections were identified in children. Septic shock was diagnosed in 6/102 (6%) cases; hypovolemic shock (due to fluid loss) was diagnosed in 2/102 (2%); a secondary infection was identified in only 1/6 (17%) patient with septic shock. Furthermore, 2/8 (25%) of the pediatric patients were diagnosed with hand-foot and mouth disease prior to the isolation of an NTS organism from the blood.

### Outcome of invasive non-typhoidal *Salmonella* infections

Overall 66/102 (65%) patients improved or recovered before hospital discharge; four (4%) died in hospital and 22 (22%) were discharged to die at home; the remaining 10 patients had an outcome that was non-assessable (five left against medical advice, two were unchanged and three transferred hospitals). One child (1/8, 12%), who was HIV-uninfected, died. The overall mortality rate was 26/92 (28%), of which 24 (92%) were HIV-infected. A total of 23% (6/26) of fatal cases had a secondary infection in BS or CSF. The median time to death in hospital was one day (IQR: 1–2 days) while median length of hospital stay for patients not discharged to die at home was 10 days (IQR: 3–15 days).

Hematology parameters for the 102 patients stratified by outcome are shown in Table 2. Notably, total white cell count was generally low (median 5.1 (IQR: 3.1–10.8) x 10^3^ cells/μl) but characterized by a high proportion of neutrophils: 82% (IQR: 66.1–87.5). The platelet count was lower in fatal cases than nonfatal cases but this was not statistically significant. Fatal cases were significantly more likely to have s higher potassium, lower hemoglobin and lower hematocrit levels (Table 2). Additionally, we performed univariable and multivariable logistic regression analyses to assess the clinical and laboratory variables that were associated with death (Table 3). Though HIV positivity, age and infecting serovar were associated with death in the univariable analysis, after controlling for confounding only HIV positivity remained independently associated with an increased risk of fatality (Table 3).

**Table 2:** Laboratory results of invasive non-typhoidal *Salmonella* disease stratified by outcome.

Laboratory test	Normal range	Total cases (n = 102)	Fatal (n = 26)	Nonfatal (n = 66)	*p* value ^a^
		Median (IQR)	Median (IQR)	Median (IQR)	
White blood cells (10^3^ cells/μl)	3.7–11.7	5.1 (3.1–10.8)	4.5 (2.9–6.5)	6.9 (3.0–11.1)	0.210
Neutrophils (%)	39.6–78.4	82 (66.1–87.5)	84.7 (74.05–88)	77.6 (63.8–85.3)	0.479
Lymphocytes (%)	14.1–52.8	11.2 (4.8–19.2)	8.05 (4.8–18.4)	12.4 (5.6–20.3)	0.261
Platelets (10^9^ cells/μl)	172–440	142 (58–258)	90 (59–153)	178 (64–269)	**0.042**
Hemoglobin (g/dL)	12.0–17.2	10.2 (7.9–12)	8.1 (6.95–10.1)	10.8 (8.9–12.5)	**0.001**
Hematocrit (%)	34.8–50.9	30.1 (23.6–36.1)	25.2 (20.8–30.4)	31.9 (26.8–37.6)	**0.002**
AST (U/L)	5–30	100.4 (52.4–189)	109 (52.4–320)	90.5 (48–133)	0.094
ALT (U/L)	5–30	47 (26–93)	36 (22–74)	47 (27.5–84.4)	0.547
Creatinine (μmol/L)	80–130	68 (57–107)	74.5 (49.5–172)	68 (55–87)	0.303
Sodium (mmol/L)	135–145	130 (126–134)	130.9 (125–135.5)	130 (127–134)	0.900
Potassium (mmol/L)	3.5–5.0	3.7 (3.3–4.5)	4.3 (3.6–4.8)	3.6 (3.1–3.9)	**0.003**

^a)^ Derived using Kruskal-Wallis test; normal values derived from minimum and maximum adult values (>18 years) listed in [42,43].

**Table 3:** Covariates associated with fatal outcome in 102 patients with invasive non-typhoidal *Salmonella* disease.

Characteristic	Univariable			Multivariable		
	OR	95%CI	p	aOR	95%CI	p
Male sex	1.13	0.42–2.99	0.813	0.82	0.23–2.95	0.757
Age category (yr) ^a^						
<10	0.75	0.07–7.88	0.811	0.94	0.06–14.3	0.967
21–30	1.75	0.44–6.88	0.423	0.31	0.05–1.85	0.198
31–40	4.32	1.20–15.6	0.025	0.86	0.15–5.0	0.866
>40	1.00	-	-	1.00	-	-
HIV infected	8.31	1.8–38.1	0.006	7.89	1.10–56.3	0.039
Hepatitis	1.36	0.50–3.71	0.552	0.89	0.23–3.44	0.862
Serovar						
*S*. Enteritidis	3.08	0.76–12.4	0.114	2.91	0.65–12.9	0.161
*S*. Typhimurium	4.24	0.91–19.8	0.066	3.24	0.54–19.5	0.198
Other	1.00	-	-	1.00	-	-

OR: odds ratio; CI: confidence interval; aOR: adjusted odds ratio

^a)^ No patients aged 10–20 years

### Treatment of invasive non-typhoidal *Salmonella* infections

The vast majority of iNTS patients received an antimicrobial (100/102; 98%) (Table 4). The most commonly used antimicrobial was ceftriaxone; 89/100 (89%) patients received this drug in mono or combination-therapy. A fluoroquinolone (levofloxacin, ciprofloxacin or ofloxacin) was used in 22/100 (22%) of cases, again either used in monotherapy or in combination with ceftriaxone (Table 3). Switching to an alternative antimicrobial (imipenem or meropenem) occurred on two occasions, of which one patient had a positive outcome and one was fatal. Trimethoprim-sulfamethoxazole was used in early therapy in 25/101(25%) iNTS; ceftriaxone was later added to this regime. Patients who were additionally diagnosed with *Talaromyces marneffei or Cryptococcus neoformans* in their BS or CSF were also treated with antifungal drugs. There was no significant difference in disease outcome with differing antimicrobial treatment regimens (Table 4). The median time from hospitalization to the use of an antimicrobial was 2.9 days (IQR 0–3 days); patients with a fatal outcome received an antimicrobial significantly earlier than those with non-fatal disease with a median of two days after hospitalization in the fatal group compared to 3.5 days in the non-fatal group (*p* = 0.01; Kruskal-wallis test).

**Table 4:** The characteristics of antimicrobial treatment for invasive non-typhoidal *Salmonella* patients

Antimicrobial prescribed	Count	Treatment, days		FCT, days ^a^	
	n/Total (%)	n	Median (IQR)	n	Median (IQR)
Any	100/102 (98)	98	7 (3–10)	51	3 (2–8)
Ceftriaxone	89/100 (89)	89	7 (4–10)	47	3 (2–7)
Monotherapy	34/100 (34)				
Combination	55/100 (55)				
Fluoroquinolone	22/100 (22)	22	10 (7–14)	11	5 (2–10)
Monotherapy	7/100 (7)				
Combination	15/100(15)				
Combination therapy	53/100 (53)				
CRO-FLQ	16/100 (16)	15	10 (7–12)	9	4 (2–5)
CRO-SXT	25/100 (25)	25	6 (2–10)	6	2 (2–5)
Other	12/100 (12)				
Switched to a broader spectrum	4/101 (4)				

FCT: fever clearance time;

^a)^ non-fatal cases only;

CRO-FLQ: ceftriaxone-fluoroquinolone; CRO-SXT: ceftriaxone- trimethoprim-sulfamethoxazole

### Serovars associated with invasive non-typhoidal *Salmonella* infections

**Fig 1:**
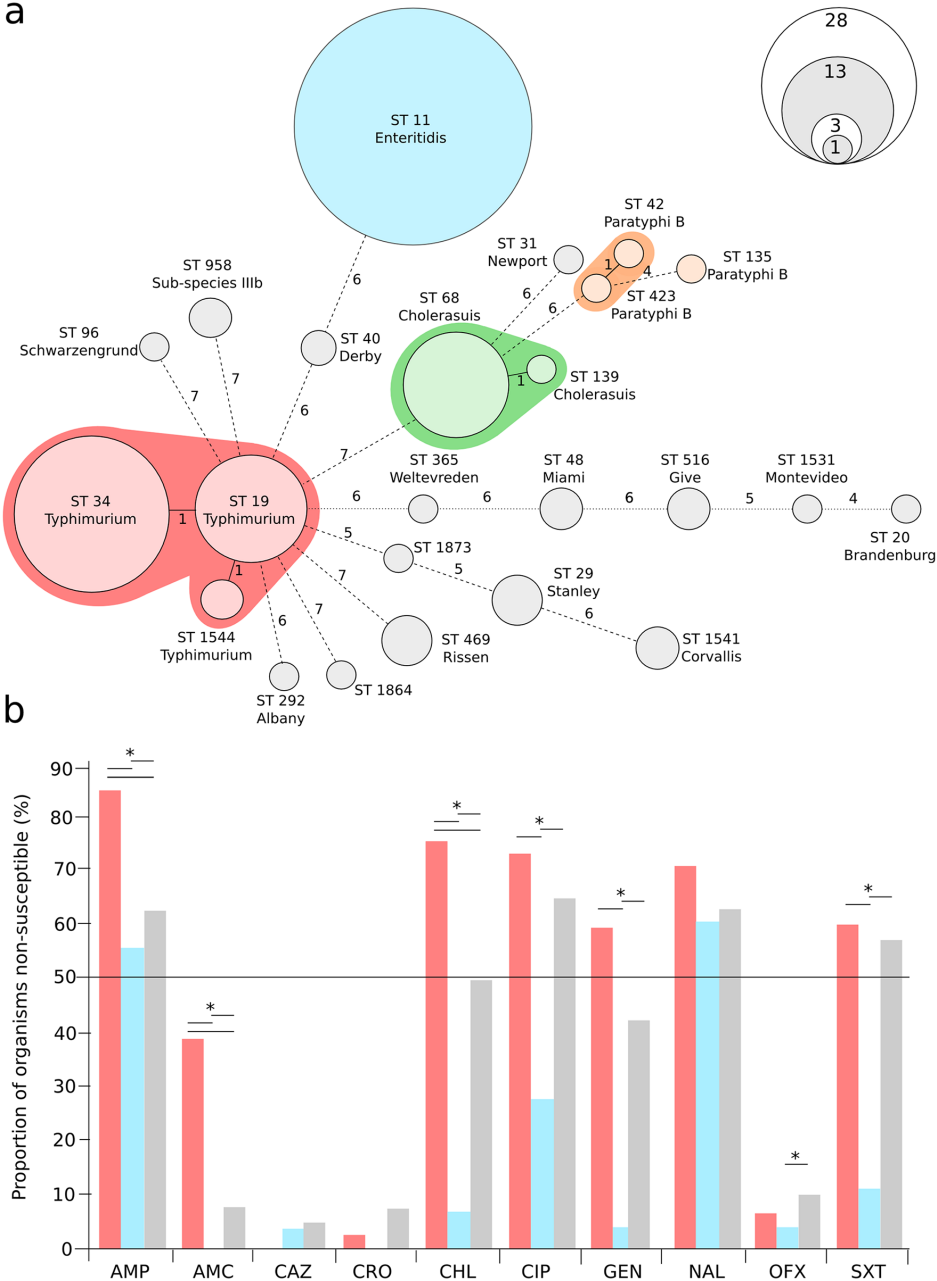
Identified *Salmonella* sequence types and serovars causing invasive disease and their antimicrobial susceptibility profiles. a) Minimum spanning tree of 142 iNTS isolates created using seven allele MLST profiling. The sequence type (ST) of each allele profile is shown along with the inferred serovar. Clonal complexes (*S*. Typhimurium, *S*. Enteritidis, *S*. Cholerasuis and *S*. Paratyphi B [tartrate positive]) are highlighted. The size of each ST group corresponds with the number of isolates with the same ST profile (scale shown), and the branches are labeled by the number of variable alleles between STs. b) Bar graph showing the proportion of organisms (red, *S*. Typhimurium; blue, *S*. Enteritidis and grey, others) exhibiting resistance against ampicillin (AMP), amoxicillin/clavulanate (AMC), ceftazidime (CAZ), ceftriaxone (CRO), chloramphenicol (CHL), ciprofloxacin (CIP), gentamicin (GEN), nalidixic acid (NAL), ofloxacin (OFX) and trimethoprim-sulfamethoxazole (SXT). Asterisks signify statistically significant differences in the proportion of organisms exhibiting resistance to the individual antimicrobial (*p*<0.05, Fisher’s exact test).

We performed MLST on the complete collection of 142 iNTS isolates cultured in this center between January 2008 and June 2013; the resulting minimum spanning tree of these data is shown in Fig 1a. We were able to identify 17 different serovars by MLST that were associated with iNTS disease in this population. The most common serovars causing invasive disease were *S*. Enteritidis (ST11) and *S*. Typhimurium (STs 19, 34 and 1544), which were responsible for 63/147 (43%) and 44/147 (30%) of all cases, respectively. *S*. Typhimurium was identified more frequently in HIV-infected patients (Table 1) (*p* = 0.003, Fisher’s exact test). The remaining organisms (n = 40) were a combination of less commonly isolated *Salmonella* serovars including *S*. Choleraesuis (n = 14), *S*. Stanley (n = 3) and *S*. Weltevreden (n = 1) (Fig 1a), which were generally identified in nonfatal cases (Table 1).

We next compared antimicrobial susceptibility profiles between *S*. Enteritidis, *S*. Typhimurium and the remaining serovars (Fig 1b). The susceptibility profiles varied between the three different etiological groups and we found that *S*. Typhimurium were significantly more likely to exhibit resistance against ampicillin, amoxicillin, chloramphenicol and trimethoprim-sulfamethoxazole than *S*. Enteritidis and the remaining serovars (*p*<0.05 for all pairwise comparisons, Fisher’s exact test) (Fig 1b). Further, >50% of *S*. Typhimurium isolates were resistant to 6/10 antimicrobials tested (including ciprofloxacin and gentamicin); the same was true for 2/10 tested antimicrobials with the *S*. Enteritidis isolates and 5/10 antimicrobials with the other iNTS isolates. The majority of iNTS were susceptible to both azithromycin and third generation cephalosporins, with the exception of a single Extended Spectrum Beta Lactamase (ESBL) producing *S*. Choleraesuis. After PCR amplification and sequencing we found this particular *S*. Choleraesuis isolate to harbor a *bla*_CTX-M-55_ ESBL gene. Despite differences in AMR profiles we found no significant difference in mortality between those infected with *S*. Typhimurium and *S*. Enteritidis (*p* = 0.431, Fisher’s exact test). Lastly, isolates from HIV–infected patients were significantly more likely to exhibit reduced susceptibility against trimethoprim-sulfamethoxazole (28/62 45%) compared to HIV-uninfected patients (6/28, 21%) (*p* = 0.037, Fisher’s exact test).

## Discussion

NTS pathogens are a leading cause of community acquired bloodstream infections in parts of sub-Saharan Africa [3,4]. In sub-Saharan Africa the disease is concentrated in children and HIV-infected adults and complicated by the recent emergence and dominance of ST313, a multidrug resistant (MDR) variant of *S*. Typhimurium [25]. It was not known whether similar epidemiological patterns and iNTS sequence types existed in Asia. We report that iNTS infections are not as common in this setting in comparison to parts of sub-Saharan Africa, but similarly the disease is associated with immunocompromised adults and primarily caused by the serovars *S*. Enteritidis and *S*. Typhimurium.

Recent evaluations in sub-Saharan Africa have highlighted that the emergence of iNTS has been largely driven in adults by the HIV epidemic, while malnutrition and malaria infection are heavily associated with iNTS in children [3,4]. The overall incidence of iNTS infections in Southeast Asia is limited compared to that of Africa [26,27], though similar to the African context we confirm that HIV infection is the primary risk factor for iNTS disease in adults in Vietnam. The overall prevalence of HIV infection is low in Vietnam (0.5%) [28], yet it is known that disease is common in adults using intravenous drugs [29]. Indeed, HIV positive individuals in our study were likely to be male between the ages of 28–37 years. Therefore, iNTS disease should be considered as a possible etiology for febrile HIV-infected individuals.

Through MLST testing we found that approximately 75% of iNTS organisms were either *S*. Enteritidis or *S*. Typhimurium, which is consistent with the organisms causing iNTS disease in Africa [5,6], and the predominant organisms found in non-invasive NTS infections in this setting [30]. Although *S*. Typhimurium isolates were more likely to exhibit resistance against antimicrobials than other serovars, we did not identify the MDR *S*. Typhimurium clone ST313, which appears to have replaced resident NTS strains in sub-Saharan Africa [25]. The sequence types identified in our setting, namely *S*. Enteritidis ST11 and *S*. Typhimurium ST19 and ST34, have been found in invasive infections in Africa previously [5,25,31,32] while *S*. Typhimurium ST1544 has recently been isolated from food samples from China [33]. We additionally identified 14 *S*. Choleraesuis isolates, a serovar known to be associated with the consumption of pork products [34], and previously shown to be a cause of bacteremia in Taiwan [35]. We surmise that it is likely that organisms causing gastroenteritis in immunocompetent individuals may be comparable to those causing iNTS disease in immunocompromised patients in Vietnam. Continuing efforts to improve food safety and hygiene may have a positive effect on reducing both non-invasive and iNTS disease in our setting, though such interventions are costly and may be difficult to sustain in an industrializing setting like Vietnam [36].

Over one quarter of patients with iNTS disease either died in hospital or were discharged to die at home with family. This mortality rate is similar to the African context and confirms the severity of this infection in an immunocompromised population. The primary risk factor for death in our population was HIV infection, confirming trends identified in adults in sub-Saharan Africa [3,4]. Though we did not have CD4 cell counts available, iNTS disease is known to be a major risk factor for death in patients with advanced HIV disease [37]. As only 22% of HIV-infected patients were on active ART at the time of admission, improving access to ART would likely prevent the number of iNTS cases in Vietnam.

The majority of patients received ceftriaxone either in mono or combination therapy. Current susceptibility profiles confirm this is an appropriate choice, however high existing resistance against a variety of antimicrobials including ampicillin, chloramphenicol and ciprofloxacin signal the propensity for Salmonellae organisms to acquire a variety of resistance mechanisms. High levels of antimicrobial resistance in *S*. Typhimurium is cause for concern, particularly as HIV-infected patients were most often diagnosed with this serovar and the presence of resistance could further complicate management. Attempting to identify whether such antimicrobial resistance is related to food consumption and the excessive use of antimicrobials in animal husbandry known to occur in Vietnam is warranted [38]. Furthermore, the significant elevation of trimethoprim-sulfamethoxazole resistance amongst HIV-infected patients suggests that pneumocystis prophylaxis with the drug leads to colonization by resistant organisms. These data indicate that reduced antimicrobial susceptibility may not purely arise in animals in zoonotic pathogens, further work regarding the use of specific antimicrobials is animals is justified.

Our study has several limitations. First, children with HIV are generally referred to one of two large local pediatric hospitals so it is likely the burden of iNTS disease in children substantially underestimated. Though HIV is a risk factor for iNTS in children in Kenya [39], malnutrition and malaria infection are also important risks in children in the sub-Saharan African context [9,40]; future work in an Asian context should examine the epidemiology of pediatric iNTS more thoroughly. Secondly, our retrospective analysis for risk of death may be biased by misclassification as we coded patients who were taken home by family members as fatal, though we did not have a confirmed death report from these individuals. Notwithstanding these limitations of a retrospective study our work provides the largest description to date of iNTS patients to date in Southeast Asia and highlights important similarities and differences between the African and Asian settings. We suggest that continued surveillance, including sequence typing/whole genome sequencing, should be performed to monitor for emergence or introduction of MDR strains or strains with any apparent enhanced virulence phenotype, such as ST313 [41].

We conclude that iNTS disease is a severe infection in Vietnam, with a mortality rate (26%) similar to that of sub-Saharan Africa. We also highlight HIV infection as the major risk for both infection and death in this setting. Though the sequence types of iNTS organisms identified in this study are common globally, we suggest continued surveillance to monitor for the presence of MDR sequence types, such as ST313, which has not, as of yet, been identified in Asia.
